# Improving Postoperative Care Through Mindfulness-Based and Isometric Exercise Training Interventions: Systematic Review

**DOI:** 10.2196/34651

**Published:** 2022-06-10

**Authors:** Allie Reynolds, Alireza Hamidian Jahromi

**Affiliations:** 1 Biological Sciences Division University of Chicago Medicine Chicago, IL United States; 2 Department of Plastic and Reconstructive Surgery Temple University Medical Center Philadelphia, PA United States

**Keywords:** postoperative care, mindfulness, isometric exercise, mindfulness-based interventions, meditation, cognitive therapy, improving care, postoperative, systematic review

## Abstract

**Background:**

Mindfulness-based cognitive therapy and isometric exercise training (IET) interventions are relatively new approaches to maintain physical functioning, alleviate pain, prevent joint stiffness and muscular atrophy, and positively influence other postoperative care outcomes.

**Objective:**

The aim of this review was to identify the impacts of mindfulness-based interventions (MBIs) and IET and, more specifically, their combination, which have not previously been assessed to our knowledge.

**Methods:**

Studies were identified by searching the PubMed and Cochrane databases within the PRISMA (Preferred Reporting Items for Systematic Reviews and Meta-Analyses) algorithm format and using relevant keyword combinations, which resulted in 39 studies meeting the inclusion criteria.

**Results:**

In general, MBI was shown to positively impact both pain relief and physical functioning, while IET positively impacted physical functioning. Numerous other benefits, including improved quality of life and decreased postoperative opioid use, were also described from both interventions; however, further research is needed to confirm these findings as well as to determine other possible benefits. No studies were found that combined MBI and IET.

**Conclusions:**

Despite many positive results from each individual intervention, there is a lack of information about how the combination of MBI and IET might impact postoperative care. The combination of these two interventions might prove to be more effective than each individual intervention alone, and the findings from this review show that they could even be complementary. Going forward, research should be expanded to study the possible benefits of the combination of MBI and IET in postoperative care routines as well as other possible combinations.

## Introduction

Postoperative care routines are particularly important in determining the long-term outcomes of many surgical procedures. Occupational therapy and physical therapy are examples of postoperative care with proven utmost importance not only in situations where the musculoskeletal system is the primary focus of the surgery but also in other surgeries on the breast, abdomen, genital, cardiovascular, and pulmonary systems, as well as other organs [1-3]. Numerous postoperative interventions have been tested in different clinical settings designed to maximize recovery or functioning, alleviate pain, prevent joint stiffness and muscular atrophy, and improve mental capabilities and coordination [4,5]. Recently, two intervention types have grown in popularity: mindfulness-based cognitive therapy (MBCT) [6] and isometric exercise training (IET) interventions [7].

Patients naturally feel stressed before surgery and during recovery. MBCT is employed as a group-based intervention, combining mindfulness meditation trainings with cognitive behavioral therapy elements [6]. Although originally used to prevent relapse in patients with depression, MBCT employed in postoperative settings is used to address preoperative anxieties, and may also influence physical functioning and overall pain relief. Patients who had higher mindfulness scores also had lower pain levels after hysterectomy procedures [8] and hand surgeries [9], demonstrating a direct relationship between mindfulness and postoperative pain relief. A different study suggested that only certain facets of mindfulness, such as the ability to describe internal experiences and to act with awareness, may be the factors contributing to optimizing psychological and physical functioning postoperatively [10]. Further research into MBCT and its impact postoperatively is needed to confirm these findings. Regardless, there is clearly a foundation in the literature surrounding mindfulness techniques and postoperative outcomes.

IET interventions are used similarly to mindfulness-based interventions (MBIs) regarding pain relief, but may be more influential in postoperative physical functioning. IET is performed by increasing muscle tension while preventing joint motion, most often by providing unmoving resistance during an exercise [11]. A meta-analysis/systematic review of 33 randomized controlled trials showed that exercise interventions can improve pain, stiffness, muscle strength, maximal oxygen uptake, and position sense (awareness) [7]. Previous research also shows that breast cancer patients who participated in a brief IET intervention showed alterations in tumor tissue gene expression [12], suggesting that exercise may have direct effects on biological mechanisms associated with cancer development and progression. IET might also influence other postoperative outcomes and their effects could be bolstered by their combination with MBI, although further research is needed.

Since MBI is more centered around the mental aspects of postoperative recovery and IET around the physical aspects of postoperative recovery, it is hypothesized that the combination of these interventions may result in even more positive postoperative results in comparison to the results observed when used individually. While other postoperative interventions are also used (which are briefly mentioned in the Discussion section), we decided to focus solely on MBI and IET for simplicity, and as an overarching example of the importance of combining mental and physical interventions in postoperative settings. Future research should expand upon this review and include other intervention types in varying combinations compared with the individual physical and mental interventions.

Thus, the aim of this systematic review was to examine the currently published medical literature on MBI and IET, and evaluate the impact of such interventions overall and specifically the overarching benefits of their inclusion in the postoperative care setting.

## Methods

**Figure 1:**
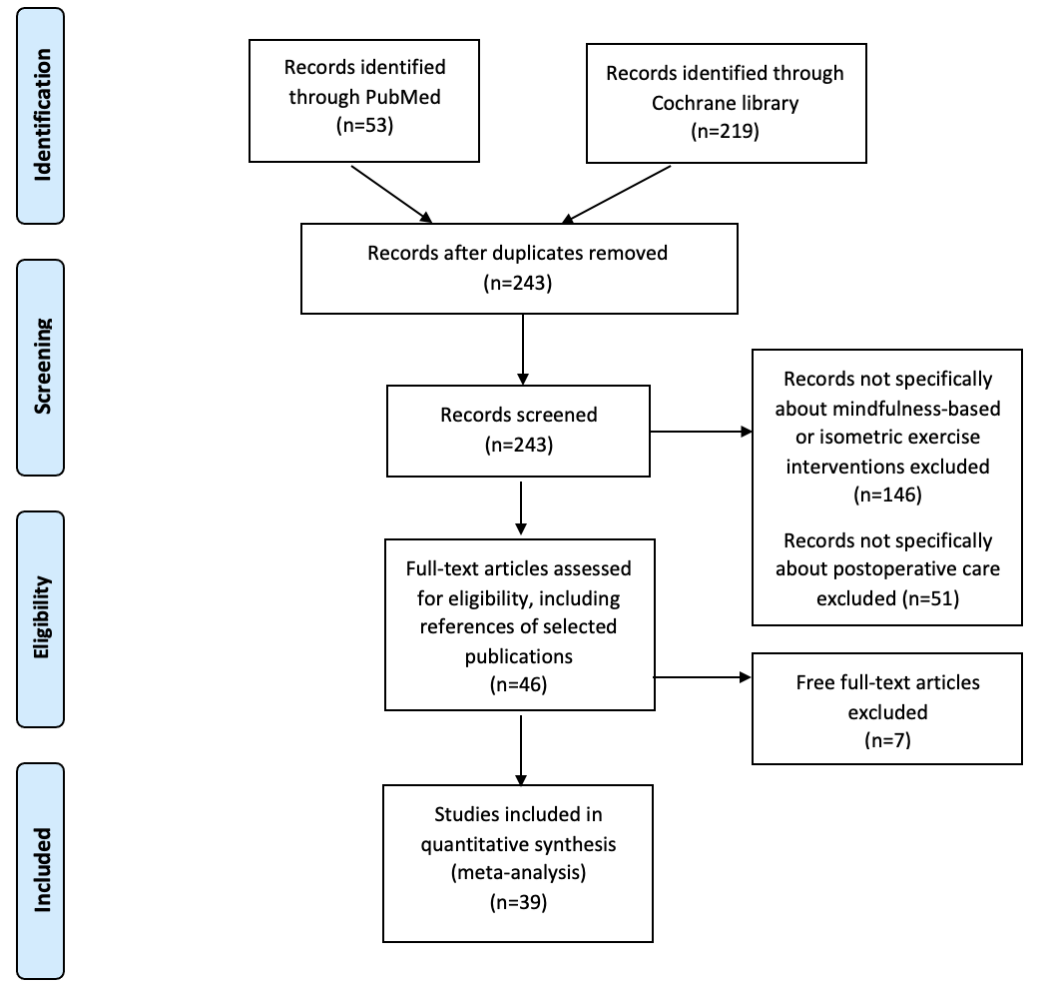
Search strategy for our systematic review to find the currently published medical literature describing usage of mindfulness-based interventions or isometric exercise interventions in postoperative care settings.

This systematic review implemented an algorithmic approach to review all of the currently available English medical literature on MBI or isometric exercises in the setting of postoperative care using the PRISMA (Preferred Reporting Items for Systematic Reviews and Meta-Analyses) principles (Figure 1). A comprehensive search of the medical literature in the PubMed and Cochrane databases was performed by one author (AR) on September 14, 2021, using the key words “mindfulness” AND “postoperative” OR “isometric exercise” AND “postoperative.” The search string was generated and the records that were not specific to MBI or IET were excluded. Articles published in a language other than English were not eligible for inclusion. No date restriction was applied. Titles and abstracts were screened by one author (AR), followed by assessment of full-text articles for eligibility and inclusion. The senior author (AHJ) supervised the process to prevent bias and checked the references. On initial and secondary searches, papers lacking a specific focus on postoperative care or those without an accessible full-text article were excluded. For completion of the search, the references of the selected publications were additionally screened with the same inclusion criteria mentioned above. The quality of the papers was assessed using the ROBINS-I (Risk Of Bias In Non-randomized Studies-of Interventions) risk of bias tool with the results reported in Multimedia Appendix 1. Only papers with low overall bias were included in this study.

## Results

### Characteristics of Included Articles

We finally included 39 full manuscripts that met our inclusion criteria. Table 1 and Table 2 define the characteristics of the final selected papers for each intervention type separately. Currently ongoing trials were not included in the final analysis. As no papers were found that combined MBI and IET outcomes, results from each category are discussed separately.

**Table 1:** Characteristics of the studies reported on the use of a mindfulness-based intervention (MBI) in postoperative (PO) care settings and the outcomes of such interventions.

Reference	Study design	Surgery	Patients, N	Intervention(s)	Duration of intervention(s)	PO outcomes
Hanley et al [13]	Randomized controlled trial	Total joint arthroplasty	118	MF^a^ of breath (MoB), MF of pain (MoP), or CB^b^ pain psychoeducation	One 20-min session 3 weeks before surgery	MoP decreased PO pain intensity and interference; MoB and MoP decreased PO opioid use
Weekes et al [14]	Randomized controlled trial	Arthroscopic rotator cuff repair	146	Relaxation exercise and control	One 5-min video and educational pamphlet	No difference in PO pain or physical function, but MBI decreased narcotic consumption at 2 weeks
Hanley et al [15]	Randomized controlled trial	Total joint arthroplasty	285	MF meditation, hypnotic suggestion, or CB pain psychoeducation	One 15-min session	MBI decreased preoperative opioid desire and increased PO physical function
Shao et al [16]	Randomized controlled trial	Breast cancer surgery	144	MBI or control	One 20-min session 5 days/ week for 6 weeks	MBI decreased PO depressive and sleep disorder symptoms
Linshaw et al [17]	One-group pretest-posttest	Mastectomy or lumpectomy for breast cancer	11	Stress Management and Resiliency Training–Relaxation Response and Resiliency Program (SMART-3RP)	8-week course	MBI improved sleep and anxiety/depression scores
Chavez et al [18]	Nonrandomized controlled trial	Lumbar spine surgery	48	Preoperative MF-based stress reduction training or control	At least one 2.5-hour class; up to 8 classes	MBI improved PO physical function and lowered system-pain interference
Haisley et al [19]	Randomized controlled trial	Minimally invasive foregut surgery	52	Virtual reality meditation/MF sessions or standard care	6 sessions	MBI patients reported higher satisfaction, and lower PO pain, anxiety, and nausea scores
Dowsey et al [20]	Randomized controlled trial	Total joint arthroplasty	127	MF-based stress reduction program or treatment as usual	8-week program	MBI improved PO pain and physical function
Yi et al [21]	Nonrandomized controlled trial	Lumbar spine surgery	48	MF-based stress reduction intervention or control	At least one 2.5-hour class; up to 8 classes	MBI group reported less PO pain but there was no difference in prescription opioid drug use
Stoerkel et al [22]	Randomized controlled trial	Breast cancer surgery	100	Treatment as usual or treatment with a “self-care toolkit”	Minimum of one listening per audio file (7 total)	MBI improved scores of pain interference, fatigue, and satisfaction with social roles. The MBI group also had less PO pain, lower erythrocyte sedimentation rate, and reduced anxiety scores
Pruthi et al [23]	Randomized controlled trial	Breast cancer surgery	29	Wearable EEG^c^ MF sensing headset device and control	3 min every day for 3 months	No differences in quality of life, fatigue, and stress, but MBI group reached outcomes sooner and had higher satisfaction
Xu and Liao [24]	Randomized controlled trial	Hip fracture fixation	100	MF-CB intervention and control group	90-min sessions 1-2 times per week	MBI group had higher general self-efficacy and lower self-perceived burden scores
Kiran et al [25]	Randomized controlled trial	Coronary artery bypass surgery	150	Rajyoga and control	3 times/day for 10 min each for 5 days	Rajyoga group had lower PO anxiety and cortisol levels

^a^MF: mindfulness.

^b^CB: cognitive behavioral.

^c^EEG: electroencephalography.

**Table 2:** Characteristics of the studies reporting the use of isometric exercise training (IET) interventions in postoperative care settings and the outcomes of such interventions.

Reference	Study design	Surgery	Patients, N	Intervention(s)	Duration of intervention(s)	Postoperative outcomes
Tapia et al [26]	Randomized controlled trial	Autologous arteriovenous fistula for hemodialysis in the upper limbs	60	IET or control	8 weeks	IET showed an increase in hand grip and better main Doppler ultrasound maturation measurements
Tapia et al [27]	Randomized controlled trial	Hemodialysis	27	IET or control	8 weeks	IET showed an increase in hand grip and clinical and Doppler ultrasound maturation measurements
Taufik et al [28]	Randomized controlled trial	Nonarticular tibia fracture	32	IET and ROME^a^ or ROME only	IET and ROME: 3 times per day; ROME: 1 time per day	IET showed higher mean bone-specific alkaline phosphatase levels and lower Hummer scale callus scores
Hong and Lee [29]	Case study	Total knee replacement	1	VR^b^ training with ROME, IET, and PT^c^	One 60-min session	Improved muscle strength, proprioception, balance, and gait ability
Auerbach et al [30]	One-group pretest-posttest	Heart transplantation	36	IET and control	One 3-min session	IET group had reduced/unchanged Doppler aortic flow parameters
Sisk et al [31]	Randomized controlled trial	Anterior cruciate ligament reconstruction	24	ES^d^ and IET or IET alone	IET: 3 times a day for 6 weeks; ES: 8 hours a day, 7 days per week for 6 weeks	No difference in isometric quadriceps strength
Huikuri et al [32]	Randomized controlled trial	Aortic valve replacement	26	Chronic aortic regurgitation and control	IET handgrip test before and after surgery	Left ventricular mass regression was smaller in patients with the most depressed ventricular responses to preoperative exercise
Huikuri and Takkunen [33]	Nonrandomized controlled trial	Mitral valve surgery	28	Groups based on mean mitral valve pressure increase during IET (>4 mmHg or ≤4 mmHg)	IET handgrip test before and after surgery	Positive correlation between the change in mean mitral valve pressure gradient during IET and changes in left ventricular functioning during exercise
Huikuri et al [34]	Randomized controlled trial	Mitral valve replacement	24	Mitral regurgitation and control	IET handgrip test before and after surgery	Positive correlation between ejection fraction changes preoperatively and postoperative resting ejection fraction changes
Huikuri [35]	One-group pretest-posttest	Mitral valve replacement	11	Mitral regurgitation	IET handgrip test before and after surgery	Improved ventricular function after surgery and left ventricular response to stress caused by IET
Tapia et al [36]	Randomized controlled trial	Native vascular access maturation for chronic kidney disease	67	IET and control	8 weeks	IET showed an increase in hand grip and improved clinical and Doppler ultrasound maturation measurements
Tal-Akabi et al [37]	Randomized controlled trial	Lower limb surgery	62	High-intensity or regular-intensity strength IET	3 weeks	High-intensity IET group lifted a greater maximal lift
Martinez Carnovale et al [38]	Randomized controlled trial	Radiocephalic arteriovenous fistula maturation	36	ES and IET or IET alone	8 weeks	ES and IET group had increased clinical and Doppler ultrasonography maturation measurements
Vaegter et al [39]	Randomized controlled trial	Total knee replacement	14	Cold pressor stimulation with aerobic IET	2 sessions (before surgery and 6 months postoperative)	Association between preoperative exercise–induced hypoalgesia and postoperative pain relief
Shaw et al [40]	Randomized controlled trial	Anterior cruciate ligament reconstruction	103	IET and control	Every day for 2 weeks	IET improved knee flexion and extension range, reduced symptom scores and sports-related postoperative problems, and lower incidence of abnormal knee laxity
Sashika et al [41]	Randomized controlled trial	Total hip arthroplasty	23	IET and ROME or control	6 weeks	IET improved maximum isometric torque on both hip sides, gait speed, and cadence
Rosenfeldt et al [42]	Randomized controlled trial	Cardiac surgery	117	IET and relaxation or control	30 min IET and 20 min relaxation 3 times per week for 2 weeks	No significant changes in quality of life, rates of postoperative atrial fibrillation, or length of hospital stay

^a^ROME: range of motion exercise.

^b^VR: virtual reality.

^c^PT: physical therapy.

^d^ES: electrical stimulation.

### Impacts of MBI

Many of the papers included in this aspect of the review cited two main benefits of MBI use: pain relief (as measured through pain medication use) and improvements in physical functioning. In comparison to other interventions such as “hypnotic suggestion” and “cognitive behavioral pain education,” MBI decreased pain medication desire and anxiety, and increased postoperative physical function in a randomized controlled trial on total joint arthroplasty with 258 patients [15]. The study investigators delivered MBI, “hypnotic suggestion,” and “cognitive behavioral pain psychoeducation” in multiple 15-minute group sessions as part of a 2-hour preoperative education program [15]. Physical function was found to be significantly higher in patients that engaged in MBI trainings 3 months after lumbar spine surgery, and system-pain interference was significantly lower at both 3 and 12 months after the intervention [18]. System-pain interference is especially important for spine surgeries, and methodologies that improve this aspect of recovery are highly sought after. A different study on total joint arthroplasty procedures also showed long-term improvements in pain and function after 12 months in patients who participated in an MBI centered on stress reduction [20]. These two studies collectively demonstrate that mindfulness interventions can influence long-term postoperative outcomes and may have implications for this type of care. Interestingly, one study separated mindfulness into two categories, mindfulness of breath and mindfulness of pain, and found that both categories decreased post total joint arthroplasty opioid use, but only mindfulness of pain decreased postoperative pain intensity and interference scores [13]. These results suggest that general MBI might not be as sufficient as more specific interventions focused on pain relief.

Other studies showed less conclusive results regarding the influence of MBI on pain relief and physical functioning. One study reported no difference in opioid use but decreased postoperative pain 1 month after lumbar spine surgery in the MBI patient group [21], indicating that effects may vary according to the procedure. Another study did not find differences in quality of life, fatigue, or stress following MBI postsurgery for breast cancer, but did note that the mindfulness group perceived the interventions to work better, were more satisfied with their quality-of-life outcomes, and reported higher utilizations of the mindfulness techniques during the study period [23]. While these reported outcomes could be stipulated for other postoperative care conditions, more studies with longer-term outcome analysis research are needed to draw a meaningful conclusion. Lastly, a different study reported no significant improvement in the quality of life of participants who received a 2-week period of MBI compared with those who only received the usual postoperative care [14]. The investigators stated that the intervention for such a short period of time was not sufficient to create a long-lasting impact [14]. They proposed that an increase in the duration of the intervention could result in more conclusive changes, furthering the idea that MBIs may be influential in long-term outcomes.

Besides pain relief and physical function, numerous other benefits were discussed in introducing MBI in postoperative care routines. One study found that MBI effectively decreased depressive and sleep disorder symptoms both 1 month and 3 months post breast cancer surgery [16]. Similarly, another study showed that sleep and anxiety/depression scores can also be improved in postoperative patients using MBI [17]. These two studies demonstrated that the beneficial impacts of MBI are not limited to pain relief and improving physical function, but that such interventions act on multiple levels of recovery. A particularly interesting study investigated the effects of “Rajyoga” interventions (a type of mindfulness meditation focused on teaching self-esteem via self-realization and improvement, charging the self, and positive attitudes), and found that patients in this intervention had lower anxiety and serum cortisol levels on the 2nd and 5th postoperative days [25]. Relatedly, patients who received MBI centered on cognitive behavior multiple times a week post hip fracture fixation surgery reported higher general self-efficacy and lower self-perceived burden scores [24], which may be related to decreases in depressive symptoms and improvements in pain relief.

Two studies employed newer technological innovations to introduce MBI to the postoperative care routine. The first study employed a “self-care toolkit” that consisted of “guided audio mind-body” techniques, an acupressure wristband, and a journal [22]. The researchers found significantly higher scores in pain interference, fatigue, and satisfaction with social roles; significantly smaller increases in the inflammatory marker erythrocyte sedimentation rate, C-reactive protein, and postoperative pain; and significantly reduced anxiety levels measured by validated outcome measures [22]. The numerous effects noted by these authors could be due to the combination of techniques or caused by each individual technique, but more research is needed to confirm the exact reason behind this observation. The second study took advantage of new technological innovations to combine MBI with virtual reality. Patients who participated in this postoperative care routine reported higher satisfaction as well as lower pain, anxiety, and nausea compared with those of the control patients [19]. Virtual reality has recently been increasing in use postoperatively, as discussed in the “Currently Ongoing Trials” section below, and will likely pave the way for postoperative care routines in the future.

### Impacts of IET

IET interventions were found to be utilized most commonly following cardiac and orthopedic surgeries. In fact, IET and its impact in the postoperative setting of any other surgical procedure were not discussed by any published article. Extending this type of intervention to other surgical procedures will be an important step in understanding the overall impact of IET on postoperative care in general. In the meantime, the benefits of IET will only be known for cardiac and orthopedic surgeries, which are discussed below.

Numerous studies cited in this review demonstrated that IET aids in postoperative recovery from cardiac surgeries. Doppler ultrasound maturation, which is indicative of blood flow efficiency, is an especially important measurement taken after cardiac surgeries. An older study used Doppler measurements and showed that isometric exercise is well-tolerated by postoperative heart transplant patients [30]. Several newer studies showed meaningful improvement in the Doppler ultrasound maturation measurements up to 2 months postoperatively in the group of patients undergoing IET compared with the control groups [26,27,36,38], suggesting that postoperative recovery is aided by isometric exercise. Hand grip is another indicator of postoperative recovery in cardiac surgery patients and was shown to be similarly improved by IET [26,27,36]. The Heikki V Huikuri lab of the University of Oulu in Finland studied the effects of IET and mitral/aortic valve replacement surgeries, providing numerous influential and high-quality publications on the topic. They showed that there was a positive correlation between the change in mean mitral valve pressure gradient and left ventricular functioning during IET [33], and a positive correlation between preoperative ejection fraction (EF) changes and postoperative resting EF changes, indicative of reduced ventricular response to afterload stress following IET [34]. Thus, to access the success of postoperative IET, it may be important to take preoperative measurements of cardiac function, resting EF, and ventricular response to afterload stress (EF changes) for comparison. In another study by this group, patients with the most depressed ventricular responses to preoperative isometric exercises had smaller left ventricular mass regression post aortic valve replacement [32]. Further research is needed to confirm and expand upon this. Lastly, they also showed that ventricular function and response to stress improved with a postoperative IET [35].

Similar benefits were also found when applying IET in orthopedic postoperative care settings with different measures of their effects. Patients in the IET group that underwent surgery for nonarticular tibia fractures were found to have significantly higher amounts of mean bone-specific alkaline phosphatase (indicative of improved osteoblastic activity) and lower Hummer scale callus scores, which are both correlated with shortened healing time [28]. Similarly, patients who performed straight leg raises and isometric quadriceps contractions every day for 2 weeks post anterior cruciate ligament (ACL) reconstruction showed significant improvements in knee flexion and extension, lessened symptom scores and sports-related complications at 6 months postoperation, and lowered abnormal knee laxity incidences [40]. Low-resistance IET and eccentric hip abductor exercises post total hip arthroplasty significantly improved the maximum isometric torque on both hips, gait speed, and cadence [41], furthering the notion that IET is beneficial to improving physical function. Combining IET with virtual reality and conventional physical therapy improved the patient’s muscle strength, proprioception, balance, and gait ability during their recovery from total knee replacement surgery in a recent case study [29]. Although this study only reported the results for one patient, it demonstrated that multiple postoperative care approaches can potentially be combined to maximize recovery. This study may guide the introduction of different combinations such as MBI and IET to optimize postoperative care in the future. Despite a different study that compared the combination of electrical stimulation and IET and found no significant difference in isometric quadriceps strength post-ACL reconstruction [31], combining postoperative care techniques with various combinations and regimens may be a future direction to optimize outcomes and mandate future evaluations in carefully controlled settings. A similar but more recent study combined IET with electrical stimulation post total knee replacement, and found that this technique was successful in relieving pain 6 months after surgery [39], corroborating the idea of combining techniques in creating a synergic impact in enhanced recovery.

The specific patient populations and their prerequisites may also influence the ideal targeted intervention, which could impact their postoperative outcomes. Defining such a modality may require extensive preoperative evaluations of the target group and in-depth knowledge of the expected postoperative changes and recovery demands. For example, elderly patients may have increased difficulty in recovering from surgery and with overall pain management than other patients, and thus may benefit from targeted postoperative care. For this population, IET resulted in greater maximal lift weights and therefore more improved physical functioning post lower limb surgery [37]. Similarly, a different study noted that there are significant differences in strength and recovery from surgery between competitive and recreational athletes [31], indicating the importance of patients’ backgrounds in determining postoperative outcomes. Thus, postoperative care should be tailored to individual patients to maximize results and create unanimous enhanced recovery across different patient populations.

Although most reports of postoperative IET showed beneficial outcomes, one study did not report a strong positive effect. In this study, exercises specifically tailored for relaxation were not found to influence pain scores or shoulder function after arthroscopic rotator cuff repair [42]. The only effect noted by these researchers was a decrease in narcotic consumption 2 weeks postoperatively [42], suggesting that IET may exert more influence on more intrusive surgeries such as total knee or joint replacements.

### Currently Ongoing Trials

In addition to the results discussed above, there are also numerous clinical trials employing variations of MBI or IET during postoperative care that are currently undergoing patient recruitment and/or are in the follow-up stage. Twelve examples of currently ongoing clinical trials are described in Table 3 [43-54].

**Table 3:** Characteristics of currently ongoing clinical trials on the use of a mindfulness-based intervention (MBI) or isometric exercise training (IET) in postoperative (PO) care settings.

Reference/identifier	Study design	Surgery	Intervention(s)	Outcomes to be reported
Olbrecht et al [43]	Randomized controlled trial	Nuss repair of pectus excavatum	Combining MBI and VR^a^	PO pain intensity
Coca-Martinez et al [44]	Randomized controlled trial	Valve replacement	IET, nutritional support, and emotional reinforcement	Incidence of PO complications
ClinicalTrials.gov, NCT04225169 [45]	Randomized controlled trial	Total knee replacement	Diaphragmatic MBI breathing exercise	PO pain, anxiety, and depression
ClinicalTrials.gov, NCT04167852 [46]	Randomized controlled trial	Bariatric surgery	MBI via a mobile platform	Accessibility to patients
ClinicalTrials.gov, NCT02104349 [47]	Randomized controlled trial	Spine surgery	MBI or music therapy group	PO pain
ClinicalTrials.gov, NCT04788329 [48]	Randomized controlled trial	Hand surgery	MBI training in “Prepare for Surgery, Heal Faster”; MBI in “Wim Hof Method”	PO pain intake and pain intensity
ClinicalTrials.gov, NCT04848428 [49]	Randomized controlled trial	Cardiac surgery	Web-based MBI	PO pain intake, pain intensity, pain interference, mindfulness, pain acceptance, pain-related catastrophic thoughts, and psychological well-being
ClinicalTrials.gov, NCT04855968 [50]	Randomized controlled trial	Shoulder arthroscopy	MBI via Headspace app	PO pain and opioid consumption
ClinicalTrials.gov, NCT04293249 [51]	Randomized controlled trial	Total joint arthroplasty	MBI or control (both prior to surgery)	Preoperative and perioperative PO pain intake, anxiety
ClinicalTrials.gov, NCT04518085 [52]	Randomized controlled trial	Breast cancer surgery	MBI or hypnosis	PO pain intake, fatigue, stress, biomarker levels
Packiasabapathy et al [53]	Randomized controlled trial	Cardiac surgery	Perioperative MBI	Program feasibility, PO pain, sleep, psychological well-being, cognitive function, and delirium
ClinicalTrials.gov, NCT03681405 [54]	Randomized controlled trial	Gynecological surgery	MBI or attention control	Adverse events, PO pain, sleep disturbances, and psychological distress

^a^VR: virtual reality.

## Discussion

### Main Findings

Although there are numerous benefits to MBIs and IET in postoperative care routines, namely surrounding pain relief and physical functioning, we found that none of the studies combined the two techniques. This was a particularly interesting finding, especially given the wide range of possible benefits each patient can obtain from each individual modality, and the potential synergistic impact that patients could gain from the combination of these two techniques. For example, patients exposed to both interventions might demonstrate increased pain relief (as seen from the MBI results) and physical functioning (as seen from the MBI and IET results) as well as other postoperative outcomes in comparison to exposure to only one of the interventions. Although MBI and IET use in postoperative settings has begun only recently, it is important to understand the benefits of their combination going forward to fully maximize postoperative patient care. We believe that the combination of interventions mainly focused on the mind settings (MBI) and physical-based interventions, especially those that can be used in the immediate postoperative period even with different immobilization settings (eg, IET), could make an ideal combination in the postoperative setting. Further research is needed to support this hypothesis and studies going forward should examine this combination in a well-controlled setting.

### Future Directions

As touched upon briefly, any measure that can help lower pain scores, improve mobility, or help with other postoperative outcomes should be utilized. This is especially true for higher-risk surgeries in which patients may learn via an MBCT on how to prepare for surgery, be better able to tolerate the surgical procedure’s impact, and use IET to facilitate their recovery from the surgery. In particular, these interventions may also be important for cancer patients who are recovering from surgery, since this patient group has been shown to demonstrate lower mindfulness scores than average, although with extensive variability [55]. Thus, cancer patients may be a potential target group for future interventions and to test the combination and potential synergic impact of an MBI and IET on their postoperative recovery.

Other intervention types outside the scope of this review were also found in the literature search. These include “Healing Touch” [56], hypnosis [57], art therapy [58], massage therapy [59], music therapy [60], and olfactory mental imagery [61,62]. Although these interventions utilized different methodologies from MBIs and IET, they may employ similar facets of mindfulness and/or exercise. These intervention types may be important to tailoring postoperative care to individual patients; however, further research into the impact of each modality alone or in combination in the setting of postoperative care is needed to improve our understanding of the optimal postsurgery care options in particular patient groups for any specific type of procedure.

As technology continues to advance, it is also important for postoperative care interventions to keep up with new innovations. This was demonstrated in this review through studies that used virtual reality settings and mobile platforms to reach their participants. As technology will continue to advance in the coming years, further innovative approaches would need to be tested to find their true benefits in advancing the postsurgical care outcome. More patient populations can be reached using technological innovations, and this has become even more important during the COVID-19 pandemic as many patients were forced into home-based programs. As an example, the Perioperative Pain Self-Management (PePS) program was created to conduct cognitive behavioral therapy sessions over the phone with rural veterans who may not have had access to this type of care otherwise [63]. Similar programs will continue to grow in importance as telemedicine increases in popularity, especially in the setting of disadvantaged communities (eg, low socioeconomic groups, underserved areas, transgender communities, ethnic minorities). It is important for the postoperative care routines to follow suit, especially in those vulnerable and disadvantaged communities.

### Conclusions

It is clear from the studies discussed in this review that there are numerous benefits to including an MBI or IET in postoperative care settings. These effects notably include pain relief and physical functioning, and may be influential in determining various other long-term outcomes. However, there were no studies found to date that combined MBI and IET. This was surprising since the combination of these two interventions might prove to be more effective than each individual intervention alone, and the findings from this review show that they could even be complementary (ie, MBIs are more effective for pain relief and physical function in surgical preparation and IET in recovery for physical function). As previously noted, it is also important to tailor postoperative care to individual patients and some patients might benefit more from combining interventions. Going forward, research should be expanded to study the possible benefits of the combination of MBI and IET in postoperative care routines as well as other possible combinations.

## Appendix

Multimedia Appendix 1 ROBINS-I risk of bias tool results for papers assessed in this review.

## Abbreviations

ACL: anterior cruciate ligament
EF: ejection fraction
IET: isometric exercise training
MBCT: mindfulness-based cognitive therapy
MBI: mindfulness-based intervention
PePS: Perioperative Pain Self-Management
PRISMA: Preferred Reporting Items for Systematic Reviews and Meta-Analyses
ROBINS-I: Risk Of Bias In Non-randomized Studies-of Interventions
