# Supplementary material for: Improving Postoperative Care Through Mindfulness-Based and Isometric Exercise Training Interventions: Systematic Review
Source: JMIR Perioper Med. 2022 Jun 10;5(1):e34651. doi: 10.2196/34651 (PMC9233259; doi:10.2196/34651)
Supplement: Multimedia Appendix 1 [file periop_v5i1e34651_app1.docx]

**Multimedia Appendix 1.** ROBINS-I risk of bias tool results for papers assessed in this review.

| Reference | Bias due to confounding | Bias in participant selection | Bias in intervention classification | Bias due to deviations from intended interventions | Bias due to missing data | Bias in outcome measurement | Bias in reported result selection | Overall bias |
| --- | --- | --- | --- | --- | --- | --- | --- | --- |
| Hanley et al [13] | Low | Moderate | Low | Low | Low | Low | Low | Low |
| Weekes et al [14] | Low | Low | Low | Moderate | Moderate | Low | Low | Low |
| Hanley et al [15] | Low | Moderate | Low | Low | Moderate | Low | Low | Low |
| Shao et al [16] | Low | Moderate | Low | Low | Moderate | Low | Low | Low |
| Linshaw et al [17] | Moderate | Low | Low | Low | Moderate | Low | Low | Low |
| Chavez et al [18] | Moderate | Moderate | Low | Low | Moderate | Low | Low | Low |
| Haisley et al [19] | Low | Moderate | Low | Low | Moderate | Low | Low | Low |
| Dowsey et al [20] | Low | Moderate | Low | Low | Moderate | Moderate | Low | Low |
| Yi et al [21] | Moderate | Moderate | Low | Low | Moderate | Low | Low | Low |
| Stoerkel et al [22] | Low | Moderate | Low | Low | Moderate | Low | Low | Low |
| Pruthi et al [23] | Low | Moderate | Low | Low | Low | Low | Low | Low |
| Xu and Liao [24] | Low | Moderate | Low | Low | Low | Low | Low | Low |
| Kiran et al [25] | Low | Moderate | Low | Moderate | Low | Low | Low | Low |
| Tapia et al [26] | Low | Moderate | Low | Low | Low | Low | Low | Low |
| Tapia et al [27] | Low | Moderate | Low | Low | Low | Low | Low | Low |
| Taufik et al [28] | Low | Moderate | Low | Low | Low | Low | Low | Low |
| Hong and Lee [29] | Moderate | Low | Low | Moderate | Low | Low | Low | Low |
| Auerbach et al [30] | Moderate | Moderate | Low | Low | Low | Low | Low | Low |
| Sisk et al [31] | Low | Moderate | Low | Low | Low | Low | Low | Low |
| Huikuri et al [32] | Low | Low | Low | Low | Low | Moderate | Low | Low |
| Huikuri and Takkunen [33] | Moderate | Moderate | Low | Low | Low | Moderate | Low | Low |
| Huikuri et al [34] | Moderate | Low | Low | Low | Low | Moderate | Low | Low |
| Huikuri [35] | Moderate | Low | Low | Low | Low | Moderate | Low | Low |
| Tapia et al [36] | Low | Moderate | Low | Low | Low | Low | Low | Low |
| Tal-Akabi et al [37] | Low | Moderate | Low | Low | Low | Low | Low | Low |
| Martinez Carnovale et al [38] | Low | Moderate | Low | Low | Low | Low | Low | Low |
| Vaegter et al [39] | Low | Moderate | Low | Low | Low | Low | Low | Low |
| Shaw et al [40] | Low | Moderate | Low | Low | Low | Low | Low | Low |
| Sashika et al [41] | Moderate | Moderate | Low | Low | Low | Low | Low | Low |
| Rosenfeldt et al [42] | Low | Moderate | Low | Low | Low | Low | Low | Low |
| Olbrecht et al [43] | Low | Moderate | Low | NI^a^ | NI | Low | NI | Low |
| Coca-Martinez et al [44] | Low | Moderate | Low | NI | NI | Low | NI | Low |
| ClinicalTrials.gov, NCT04225169 [45] | Low | Moderate | Low | NI | NI | Moderate | NI | Low |
| ClinicalTrials.gov, NCT04167852 [46] | Low | Moderate | Low | NI | NI | Low | NI | Low |
| ClinicalTrials.gov, NCT02104349 [47] | Low | Moderate | Low | NI | NI | Low | NI | Low |
| ClinicalTrials.gov, NCT04788329 [48] | Low | Low | Low | NI | NI | Low | NI | Low |
| ClinicalTrials.gov, NCT04848428 [49] | Low | Low | Low | NI | NI | Low | NI | Low |
| ClinicalTrials.gov, NCT04855968 [50] | Low | Moderate | Low | NI | NI | Low | NI | Low |
| ClinicalTrials.gov, NCT04293249 [51] | Low | Moderate | Low | NI | NI | Low | NI | Low |
| ClinicalTrials.gov, NCT04518085 [52] | Low | Moderate | Low | NI | NI | Low | NI | Low |
| Packiasabapathy et al [53] | Low | Moderate | Low | NI | NI | Low | NI | Low |
| ClinicalTrials.gov, NCT03681405 [54] | Low | Moderate | Low | NI | NI | Low | NI | Low |

NI^a^: no information.
